# Structural mutants of dengue virus 2 transmembrane domains exhibit host-range phenotype

**DOI:** 10.1186/1743-422X-8-289

**Published:** 2011-06-09

**Authors:** Katherine M Smith, Kavita Nanda, Carla J Spears, Mariana Ribeiro, Ricardo Vancini, Amanda Piper, Gwynneth S Thomas, Malcolm E Thomas, Dennis T Brown, Raquel Hernandez

**Affiliations:** 1 Arbovax, Incorporated, Raleigh, North Carolina, USA; 2 Department of Molecular and Structural Biochemistry, North Carolina State University, Raleigh, North Carolina, USA; 3 Wake Forest University, Department of Molecular Pathology, Medical Center Boulevard, Winston-Salem, North Carolina, USA

## Abstract

**Background:**

There are over 700 known arboviruses and at least 80 immunologically distinct types that cause disease in humans. Arboviruses are transmitted among vertebrates by biting insects, chiefly mosquitoes and ticks. These viruses are widely distributed throughout the world, depending on the presence of appropriate hosts (birds, horses, domestic animals, humans) and vectors. Mosquito-borne arboviruses present some of the most important examples of emerging and resurgent diseases of global significance.

**Methods:**

A strategy has been developed by which host-range mutants of Dengue virus can be constructed by generating deletions in the transmembrane domain (TMD) of the E glycoprotein. The host-range mutants produced and selected favored growth in the insect hosts. Mouse trials were conducted to determine if these mutants could initiate an immune response in an *in vivo *system.

**Results:**

The DV2 E protein TMD defined as amino acids 452SWTMKILIGVIITWIG467 was found to contain specific residues which were required for the production of this host-range phenotype. Deletion mutants were found to be stable in vitro for 4 sequential passages in both host cell lines. The host-range mutants elicited neutralizing antibody above that seen for wild-type virus in mice and warrant further testing in primates as potential vaccine candidates.

**Conclusions:**

Novel host-range mutants of DV2 were created that have preferential growth in insect cells and impaired infectivity in mammalian cells. This method for creating live, attenuated viral mutants that generate safe and effective immunity may be applied to many other insect-borne viral diseases for which no current effective therapies exist.

## Background

Dengue Virus (DV), the most prevalent arbovirus, is in the family *Flaviviridae *and has four distinct serotypes which cause an acute disease of sudden onset with headache, fever, prostration, myalgia, lymphadenopathy and rash [1,2]. DV is transmitted by mosquitoes and as distribution and density of these insects has expanded, a considerable increase in Dengue transmission has been observed in tropical and subtropical areas throughout the world, with about 50 million cases of Dengue Fever and 500,000 cases of the more severe Dengue Haemorrhagic Fever (DHF). Over 20,000 deaths each year can be attributed to DHF, ranking Dengue with tuberculosis, STDs (including HIV), childhood diseases or malaria in costs of care and economic impact. DV is also the only known arbovirus that has fully adapted to the human host and has lost the need of an enzootic cycle for maintenance [1]. The lack of prophylactics, vaccines or antivirals against DV alone leaves 2 billion people at risk yearly to contract this disease [1].

**Figure 1:**
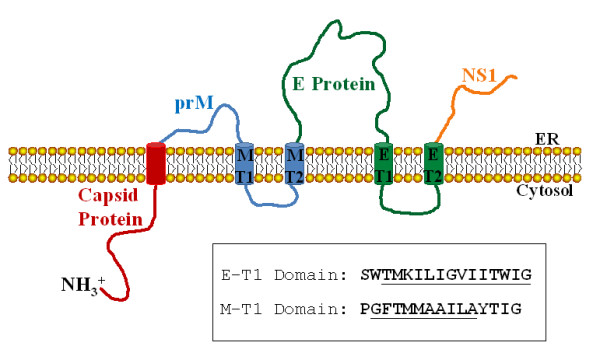
**Schematic representation of the organization of dengue virus polyprotein**. Representation of DV protein structure illustrating the predicted orientation across the endoplasmic reticulum (ER). Cylinders represent transmembrane (T) helices. prM, membrane protein precursor; E, envelope protein; NS1, non-structural protein. The sequences of the E protein T1 and M protein T1 are shown. Underlined residues indicate the amino acids targeted for deletion. The TM domain of the capsid protein is cleaved during processing and is not present in the membrane of the assembled virus.

DV is an enveloped virus with an icosahedral capsid that contains a single-stranded, positive sense RNA genome [3]. The envelope of DV is composed of hetero-dimers of the (E) glycoprotein and the membrane (M) protein that are embedded in a host-derived lipid bilayer (Figure 1). The nucleocapsid is composed entirely of capsid (C) protein and encapsulates the RNA genome. The E glycoprotein is important for cell receptor attachment and subsequent infection of the target cell membrane, and bears the neutralization epitopes [4]. DV, as well as all arboviruses, has evolved to replicate in the unique biochemical environments of both vertebrate and invertebrate hosts [5]. As a result, the mature viruses are hybrid structures which derive their lipid bilayers from the host cell. Hence, composition of the outer surface of mature dengue virions varies depending upon the type of host cell in which the virus was produced. Insect cell membranes do not contain endogenous cholesterol and are composed of shorter-chain lipids than mammalian membranes [6]. Consequently, insect cell membranes are thinner in cross-section as compared to mammalian membranes [7-10]. The membrane-spanning domains (transmembrane domains; TMD) of proteins integrated into insect cell membranes have evolved to accommodate both host membranes. However, it is hypothesized that shorter transmembrane domains of viruses can be tolerated in insect cell membranes verses mammalian membranes [11]. In Sindbis virus (SV), an arbovirus of the family *Alphaviridae*, large truncations of the E2 TMD are tolerated in insect hosts, but not mammalian cells, confirming the theory that insect cells do not require the same membrane spanning length of E2 as those integrated into mammalian membranes [11]. This host-derived TMD difference was used to develop a method for production of viral mutants with truncated TMDs that are capable of efficient growth in invertebrate cells but with impaired replication in vertebrate cells [11]. A targeted and rational method of deleting amino acids in the TMD of the envelope glycoproteins was used to create DV serotype 2 (DV2) mutants with preferential growth in the insect host (Patent No. 6,589,533). Based on the SV model, it was predicted that deleting amino acids in the TMD of the E or M proteins of dengue virus would make these domains shorter such that they would be capable of spanning an insect but not the mammalian cell membrane. This alteration was expected to result in the production of mutant virus which demonstrated reduced infectivity in mammalian hosts but retained efficient growth in insect hosts, producing a host-range phenotype. Deletions in the TMD of SV resulted in virus with altered infectivity and host-range [11]. Both E and M proteins of DV have a TMD that can be targeted for deletion mutation analysis using the SV TMD deletion strategy. In the study reported herein, mutants of DV2 were created and analysed for a host-range phenotype with preferential growth in insect cells. Mosquito-preferential DV2 host-range mutants were created by this deletion strategy; however, it was determined that other factors irrespective of TMD length also affect phenotype. Truncations of 3 to 4 amino acids in the TMD of the DV2 E domain at positions between amino acids 458 to 463 resulted in virus with attenuated growth in mammalian cells that maintained the ability to replicate in mosquito cells while larger deletions resulted in either no or very low levels of virus production and infectivity. When injected into mice, these mutants were found to elicit a higher level of neutralizing antibody than the wild-type virus and warrant further study as potential vaccine candidates.

## Methods

### Cell Culture

C6/36 cells (*Aedes albopictus*, American Type Culture Collection [ATCC] # CRL-1660, Manassas, VA) were maintained in minimal essential medium (MEM) containing Earl's salts supplemented with 10% fetal bovine serum (FBS), 5% tryptose phosphate broth (TPB) and 2 mM L-glutamine. Vero cells (African Green monkey kidney, ATCC #CCL-81) were maintained in 1X MEM supplemented with 10% FBS, 5% TPB, 2 mM L-glutamine, 10 mM Hepes pH 7.4 and 1X MEM nonessential amino acids (NEAA) (1:100 dilution of NEAA from Gibco #11140, Carlsbad, CA).

### Construction of DV2 deletion mutants

A full-length cDNA clone of Dengue serotype 2 (DV2; Thai strain 16681, GenBank # U87411) in pGEM3z+ was obtained from the Walter Reed Army Institute of Research for these studies [12]. The clone produces full-length DV2 RNAs when transcribed *in vitro *with T7 RNA polymerase and after transfection of the transcripts into mammalian or insect cells, infectious virions were generated.

Deletions in the TMD of the DV2 E and M proteins were produced by polymerase chain reaction (PCR)-based site-directed mutagenesis, using *Pfu Turbo*^® ^DNA polymerase AD (Stratagene, La Jolla, CA). Primers were designed to create sets of single, double, triple, quadruple, and quintuple amino acid (aa) deletions within the T1 domain of the E or M protein of DEN2. PCR conditions were as follows: 25 ng DV2 DNA, 1X or 1.5X *Pfu Turbo *Buffer, 0.4 mM/μL dNTPs (New England Biolabs, Ipswich, MA), 5 ng/μL each primer, and 0.1 U/μL *Pfu Turbo *DNA polymerase AD. Reactions were run with and without DMSO (4% final concentration). PCR cycles were as follows: 95°C for 2 min, then 25 cycles of 95°C for 15 sec, 45 sec of annealing (T_A _= Primer T_m_-5°C for each set of primers), 68°C for 24 min. Extension was performed for 28 min at 68°C; samples were held at 4°C until analysis by gel electrophoresis. Following mutagenesis of the WT DV2 clone, the PCR products were digested with *DpnI *(New England Biolabs) and transformed into SURE^®^2 Supercompetent *E.coli *cells (Stratagene) as per manufacturer's instructions with a few alterations. Following heat shock and recovery on ice, room temperature NZY^+ ^broth (Teknova, Hollister, CA) was added and incubation was performed at 30°C for 1 to 2 hours with shaking. After plating on Luria Broth (LB) agar containing 50 μg/mL carbenicillin (Teknova) incubation was performed at 30°C for 32 to 48 hours. A colony PCR screen [13] was then used to quickly identify the presence of the mutations in the resulting bacterial colonies prior to culture. Growth of all DV2 clones in SURE^®^2 cells was conducted in LB containing 50 μg/mL carbenicillin at 28 to 30°C for approximately 24 to 48 hours with shaking. DV2 plasmid DNA was recovered using the Wizard^® ^Plus Minipreps DNA Purification System (Promega, Madison, WI) following manufacturer's instructions. All DV2 deletion mutant clones were confirmed by sequence analysis (Eurofins MWG Operon, Huntsville, AL).

### In vitro transcription and RNA transfection

Transcripts were generated for each DV2 mutant clone using the RiboMAX™ Large Scale RNA Product Systems for T7 RNA Polymerase (Promega) following manufacturer's instructions, with the addition of RNA cap analog 7 mg(ppp)G (NEB # S1404S). The RNA transcripts were transfected into Vero and C6/36 cells as follows: Cells were pelleted and washed in RNase free electroporation buffer (PBS-D for Vero and MOPS for C6/36) and resuspended in their respective buffers at a concentration of 1 × 10^7 ^to 5 × 10^7 ^cells/ml. RNA transcripts were added to 400 μl of cells and electroporated at 1.0 KV, 50 μF and 8 resistance using the BioRad Gene Pulsar II (Bio-Rad Laboratories, Hercules, CA). The transfected cells were then plated at different concentrations in three different 24 well plates with 1.0 ml of the media and incubated at 37°C for Vero cells and 28°C for C6/36 cells for 1 hour with slow rocking. The media was removed and the plates overlayed with 1.0 ml of 1% carboxymethylcellulose (CMC) in 1X Vero media or 1X C6/36 media and incubated for 7, 10 and 14 days. The plates were developed by focus assay.

### Focus assay

The focus assay may be developed as a colorimetric or fluorescent assay using antibodies labeled with either HRPO (color substrate) or Alexa Fluor fluorescent dye. For the color assay, plates with transfected or infected cells were washed twice with 1X PBS and fixed with 80% methanol for 15 minutes at room temperature, followed by incubation with antibody dilution buffer (5% skim milk in 1X PBS-D) for 10 minutes. Primary antibody (α-DV NS1 glycoprotein, Abcam #ab41623, Cambridge, MA) was added at a dilution of 1:400 in antibody (Ab) dilution buffer and incubated for 1 hour at 37°C with slow rocking. The wells were then washed twice with 1X PBS followed by the addition of secondary antibody conjugated with horse radish peroxidase (HRP) (Sigma # 8924, St. Louis, MO) at a dilution of 1:500 in Ab dilution buffer. Wells were washed again twice with 1X PBS. Foci were visualized by the addition of 150 μl TrueBlue™ peroxidase substrate (KPL# 50-78-02, Gaithersburg, MD) to each well and developed for ~15 minutes. Foci were counted and titer determined in focus forming units/ml (ffu/ml) of virus. For the fluorescent assay, the protocol is similar to the color assay with the following exceptions: Cells were fixed for 20 minutes at room temperature in 100% methanol. A second 10 minute incubation with 1X PBS plus 0.05% Tween, followed by 2 washes with 1X PBS plus 0.2% BSA. Antibody was diluted in 1X PBS + 0.2% BSA. The washes between the primary and secondary antibodies were performed in 1X PBS + 0.2% BSA. The secondary antibody, Alexa fluor^® ^488 F(ab')_2 _fragment of goat anti-mouse IgG (Invitrogen # A-11017, Carlsbad, CA), incubation was conducted for 45 minutes in darkness. After the final wash, 50 μl of water was added to each well for visualization of the fluorescent foci.

### Primary Virus Screen

A mutant virus screen was developed to determine the infectivity of each mutant virus by visualizing and counting foci of infection [14]. The screen was performed by first transcribing the linearized mutant DV2 DNA clones into RNA as described previously [11,15]. RNA transcripts were transfected into Vero and C6/36 as explained above. The transfected cells were then plated out at different concentrations in three different 24 well plates with 1.0 ml of the media and incubated at 37°C for Vero cells and 28°C for C6/36 cells for 1 hr with slow rocking. The plates were overlayed with 1.0 ml of 1% CMC in 1X Vero media or 1X C6/36 media and incubated for 7, 10 and 14 days. Foci were visualized by color focus assay and scored. Each focus represents an infectious center demonstrating that the virus is able to infect the neighboring cells and is considered a plus in this screen.

### Secondary Screen

DV2 mutant clones that passed the first screen (i.e. demonstrated the ability to produce foci of infection), were transcribed into RNA and transfected again into C6/36 cells. The transfected cells were then transferred to 25 cm^2 ^flasks and incubated for 7 days at 28°C. The virus was collected and amplified once by infecting another flask of C6/36 cells. After amplification, virus was harvested on day 7 and titrated using either Vero or C6/36 as indicator cells. Serial viral dilutions were prepared with dilution buffer (PBS-D + 3% FBS) and 200 μl of virus. Each dilution was used to infect cell monolayers in 24 well plates for 1 hour at room temperature or 37°C. The infected cell monolayers were then overlayed with 1.0 ml of 1% CMC in 1X Vero media or 1X C6/36 media and incubated at their respective temperatures for 7 days. Foci of infection were detected by focus assay as described above. Once a measurable titer was observed for the mutant viruses, the virus was used to infect Vero and C6/36 cell lines at an MOI (multiplicity of infection) of ~0.03 ffu/cell. At day 7 post-infection, the virus was harvested and titrated as described above in order to determine the host-range phenotype. Mutants which exhibited the desired host-range phenotype were then amplified and plaque purified.

### Infection and purification of selected mutants

The WT and DV2 mutants were grown in the *Aedes albopictus *mosquito-derived C6/36 cell line. Cells were split one day prior to infection at a ratio of 1:3. Subconfluent monolayers of C6/36 cells were infected at an MOI of ~0.03 ffu/cell. Virus was diluted in C6/36 media and each 75 cm^3 ^flask infected with 1.0 ml of diluted virus for 1 hour at room temperature. After the initial infection, 4.0 ml of fresh media was added to each flask. Flasks were then incubated for 7 days at 28°C. Virus was harvested by centrifugation of the supernatant at 4000 rpm for 10 min. Purification and concentration of WT and mutant DV2 were achieved using isopycnic ultracentrifugation with iodixanol (Optiprep) gradients (Sigma, St. Louis, MO). Virus was spun to equilibrium in gradients of 12% to 35% iodixanol and isolated twice.

### Expression and Processing of DV2 mutant proteins

Analysis of the production and processing of DV2 host-range mutant proteins was done using PAGE and western blots and were done on virus from *A. albopictus *C6/36 cells. Mosquito cells were infected with WT, DV2ΔGVII, and DV2ΔLIG and incubated at 28°C for 1 week. Infected mosquito cell supernatants were then harvested and purified in iodixanol gradients as described above. Purified virus amounts were determined using an EZ-Q protein determination kit (Invitrogen, Carlsbad, CA). Protein purity was then determined. Twenty-five μl of each preparation was loaded onto 4-12% bis-tris gradient gels (Invitrogen) and was stained with colloidal Coomassie (Pierce, Rockford,IL) to determine purity. Proteins from a duplicate gel were transferred to a polyvinylidene difluoride membrane and blotted as described previously [16] with the following modifications: Primary anti-whole dengue virus mouse monoclonal antibody (Abcam #ab9202) was used for detection; an anti-mouse-HRP conjugate was used as a secondary antibody; and viral proteins were visualized by the addition of TrueBlue™ peroxidise substrate (KPL, Gaithersburg, MD) to correctly assign the E and M proteins within the virus particles.

### Preparation of virus inoculum for mouse studies

Mosquito cells (C6/36) were infected with WT, DV2ΔGVII, and DV2ΔLIG and mutants and incubated at 28°C for 1 week as described above. Infected mosquito cell supernatants were then harvested and purified twice in iodixanol gradients as described above. Infected mosquito cell supernatants were then harvested and concentrated by tangential flow filtration (TFF) using a 100 kDa cut off membrane as per manufacturer's recommendations (PALL, Post Washington, NY) and further purified in iodixanol gradients as described above. Twenty-five μl of each preparation was loaded onto a 4-12% bis-tris gradient gel (Invitrogen) to test for purity. Protein purity was determined using 4-12% bis- tris gradient gels (Invitrogen) and stained with colloidal Coomassie (Pierce, Rockford,IL) to determine purity. The inoculum mass to inject per mouse was determined from these assays. This investigation conformed to the Guide for Care and Use of Laboratory Animals published by NIH (No 82-23, rev 1996). The protocol was approved by the Institutional Animal Care and Use Committee at Immunobiosciences, Inc., our subcontractor for all animal studies.

### RT-PCR analysis of mutant viruses

To confirm that the desired deletions remained intact in virus grown in cell culture, RNA was extracted from each mutant virus, reverse transcribed, and amplified by PCR (RT-PCR). RNA extraction was performed by two methods. The first method involved extracting RNA from a minimum of 10^4 ^ffu of virus by pelleting the virus at 50,000 rpm in a SW55Ti (Beckman Coulter, Brea, CA) rotor for 1 hour. The pelleted virus was extracted as described previously [15]. The RNA pellet was resuspended in 10 μl of diethyl pyrocarbonate (DEPC) treated water and checked on 1% agarose gel. For smaller quantities of virus, a second method of purification was employed. Viral RNA was harvested from C6/36 cells by RNeasy Mini kit (Qiagen, Valencia, CA). Infected cells were scraped off flasks on Day 7 post-infection and suspended in media at a cell density of ~ 1 × 10^7 ^cells/ml. Cells were spun down, resuspended in lysis buffer, and homogenized. Viral RNA was purified as directed according to manufacturer's instructions. The resulting RNA was suspended in 30 μl of RNase free water and checked on a 1% agarose gel.

The extracted RNA was reverse transcribed and amplified by PCR using the One-Step RT-PCR kit (Qiagen). Primers were designed for use in the RT-PCR reaction by analysing the folded DV2 RNA structures to optimize RNA binding accessibility [17]. The products generated in the RT-PCR reaction (~ 640 bp) were phenol/chloroform extracted, precipitated and sequenced to confirm the identity and presence of deletions. Some of the RT-PCR products were of insufficient quantity and quality to be sequenced directly. These products were amplified by nested PCR, subcloned into the pDrive cloning vector and transformed in QIAGEN EZ Competent cells using the QIAGEN PCR cloning^plus ^kit (Qiagen). White colonies containing the vector-ligated PCR product were amplified and the minipreped DNA was sequenced for verification (Eurofins MWG Operon).

### Focus reduction neutralization test (FRNT)

The relative amount of virus-neutralizing antibody present in the mouse sera was determined by a focus reduction assay on Vero cells, which is similar to the standard Plaque Reduction Neutralization Test [1]. The assay was performed as described above for the focus assay, with the addition of a pre-incubation step to allow serum antibody to bind WT DV2. In short, serum samples were heat inactivated for 30 minutes at 56°C. A dilution of 1:10 for each sample was prepared followed by serial 2-fold dilutions in dilution buffer containing 3% FBS. Each dilution was mixed with an equal volume of virus suspension containing 50 ffu of WT DV2 and incubated at 37°C for 1 hour. 200 μl of the mixture was then added to duplicate wells seeded with Vero in a 24 well plate. After 1 hour of adsorption at 37°C, the cells were overlayed with 1X Vero media containing 1% CMC. At day 7 post-infection, antibody titers were determined by developing the plates according to the focus assay protocol. Neutralizing antibody titers (FRNT_50_) were reported as the highest dilution of the sera that reduced focus formation by 50%.

### Experimental design for determination of neutralizing antibody titers

To evaluate DV2 specific neutralizing antibody responses, five groups (n = 5) of 8 week old BALB/cJ mice were inoculated subcutaneously (SC) with 29 μg (~10^2^-10^3 ^ffu/mouse) of purified WT DV2, DV2ΔLIG, DV2ΔGVII, or iodixanol buffer alone. Protein estimates were made using the EZQ^® ^Protein Quantitation Kit (Molecular Probes). Mice were boosted with an equal dose on Day 14 and serum samples were collected from all groups on Day 28, at the termination of the study. The relative amount of virus-neutralizing antibody present in mouse sera was determined by focus reduction neutralization test as described above (12).

### Transmission Electron Microscopy

Vero or C6/36 cells were transfected with RNA transcribed from WT DV2, DV2ΔLIG, or DV2ΔGVII clones. Incubation proceeded at 37°C for 16-18 hours, after which the cell monolayers were scraped from the flasks and pelleted by low speed centrifugation. Cell pellets were washed twice with PBS and fixed with 3% glutaraldehyde (Ladd Research Industries, Inc., Williston, VT) in 0.1M cacodylic acid buffer pH 7.4 (Ladd Research Industries). After washing 3 times with 0.1M cacodylic acid, cells were stained with 2% osmium tetroxide in cacodylic buffer for 1 hour. Cells were then washed as before and embedded in 2% agarose. Agarose containing the cell sample was then pre-stained with 1% uranyl acetate (Polaron Instruments Inc., Hatfield, PA) overnight at 4°C. The samples were washed and carried through sequential dehydration with ethanol. Infiltration was achieved using SPURR compound (LADD Research Industries). Next blocks were trimmed on an LKB NOVA Ultrotome (Leica Microsystems, Inc., Deerfield, IL). Ultra-thin sections were obtained and stained with 5% uranyl acetate in distilled water for 60 minutes and in Reynolds lead citrate pH 12 (Mallinkrodt Baker Inc., Paris, KY) for 4 minutes. The samples were examined at 80 kV in a JEOL JEM 100S transmission electron microscope.

## Results

### Production of Dengue virus TMD mutants

The exact sequences or structures of Flavivirus TMDs (including DV) E proteins are not precisely known, however they can be predicted with a high degree of certainty by sequence analysis [18]. The TMDs of Flaviviruses are predicted to be shorter than those of Alphaviruses, such as SV (DV14-16 amino acids compared to SV 26-27 amino acids), based on the sequence and that the envelope membrane is derived from the endoplasmic reticulum (ER) [18,19]. This prediction is supported by physiological differences in the virus assembly. Unlike Alphaviruses that bud from a cell's plasma membrane, Flavivirus particle budding occurs from the ER [20] which is thinner and contains less cholesterol than the plasma membrane [10]. This observation is consistent with the putative shorter TMDs predicted to occur in Flaviviruses. As is found for Alphaviruses, Flavivirus TMDs have no consensus sequence but model as hydrophobic helices [21]. Based on these data, we predict that the shorter length of the Flavivirus TMDs will require fewer deletions to produce the desired host-range phenotype as observed in the Alphavirus Sindbis. DV2 has two TMDs targeted for deletion mutagenesis in this study: the first TMD of the E protein (E-T1), predicted to contain 16 amino acids, and the first TMD of the M protein (M-T1), calculated to contain 14 amino acids [3]. Deletions of 1 to 5 amino acids were created sequentially in each TMD, producing mutants containing 11 to 15 amino acids (E-T1) or 13 to 9 amino acids (M -T1) remaining in the membrane. Thirty-four DV2 E-T1 and 9 M-T1 deletion mutants were produced in DV2 (Table 1). Transcripts produced from each clone were transfected into cultured *Aedes albopictus *C6/36 (insect) and Vero (mammal) cells as described in Materials and Methods. Transfected cells were transferred to 24 well plates and incubated for 7 to 10 days at 28°C or 37°C, respectively. At the end of the incubation period, the presence of virus was visualized by focus assay.

**Table 1:** Dengue virus deletion mutants in the first TMD of the E protein of DV2

SEQUENCE E-T1 Domain			Foci		Critical AA Vero	Critical AA C6/36
					
			Vero	C6/36		
	**WT DV2**	**_452_SWTMKILIGVIITWIG_467_**	**++**	**++**		

1	ΔIG_466-67_	**SWTMKILIGVIITW--**	**-**	**-**		**2aa**

2	ΔW_465_	**SWTMKILIGVIIT-IG**	+	-		

3	ΔT_464_	**SWTMKILIGVII-WIG**	+	-		**T_464_**

4	T_464_A	**SWTMKILIGVIIAWIG**	**+**	**+**		

5	ΔIT_463-64_	**SWTMKILIGVI--WIG**	+	-		**2aa**

6	ΔIIT_462-64_	**SWTMKILIGV---WIG**	**-**	**-**		**T_464_**

7	ΔVIIT_461-64_	**SWTMKILIG----WIG**	+	-		**T_464_**

8	ΔGVIIT_461-64_	**SWTMKILI-----WIG**	+	-	**5aa**	**5aa**

9	ΔI_463_	**SWTMKILIGVI-TWIG**	+	-		**I_463_**

10	ΔII_462-63_	**SWTMKILIGV--TWIG**	+	-		**2aa**

11	ΔVII_461-63_	**SWTMKILIG---TWIG**	**-**	**-**		

12	ΔGVII_460-63_	**SWTMKILI----TWIG**	**+**	**+**		

13	ΔIGVI_459-64_	**SWTMKIL----ITWIG**	+	-		

14	ΔIGVII_459-63_	**SWTMKIL-----TWIG**	+	-	**5aa**	**5aa**

15	ΔV_461_	**SWTMKILIG-IITWIG**	**+**	**+**		

16	ΔGV_460-61_	**SWTMKILI--IITWIG**	+	-		**2aa**

17	ΔIGV_459-61_	**SWTMKIL---IITWIG**	**+**	**+**		

18	ΔLIGV_458-61_	**SWTMKI----IITWIG**	+	-		

19	ΔILIGV_457-61_	**SWTMK-----IITWIG**	**-**	**-**	**5aa**	**5aa**

20	ΔG_460_	**SWTMKILI-VIITWIG**	**+**	**+**		

21	ΔIG_459-60_	**SWTMKIL--VIITWIG**	+	-		**2aa**

22	ΔLIG_458-60_	**SWTMKI---VIITWIG**	**+**	**+**		

23	ΔILIG_457-60_	**SWTMK----VIITWIG**	**+**	**+**		

24	ΔILI_457-59_	**SWTMK---GVIITWIG**	**+**	**+**		

25	ΔIL_457-58_	**SWTMK--IGVIITWIG**	**-**	**-**		**2aa**

26	ΔI_457_	**SWTMK-LIGVIITWIG**	+	-		**I_457_**

27	ΔKI_456_	**SWTM--LIGVIITWIG**	+	-		**2aa**

28	ΔK_456_	**SWTM-ILIGVIITWIG**	**-**	**-**	**K_456_**	**K_456_**

29	K_456_A	**SWTMAILIGVIITWIG**	**+**	**+**		

30	ΔMK_455-56_	**SWT--ILIGVIITWIG**	+	-		**2aa**

31	ΔM_455_	**SWT-KILIGVIITWIG**	**-**	**-**	**M_455_**	**M_455_**

32	ΔTM_454-55_	**SW--KILIGVIITWIG**	**+**	**-**		**2aa**

33	ΔT_454_	**SW-MKILIGVIITWIG**	**+**	**+**		

34	ΔW_453_	**S-TMKILIGVIITWIG**	**+**	**+**		

Deletions in the E-T1 domain of DV2 were produced by site-directed PCR-based mutagenesis. The mutant viruses were screened by a DV2 mutant transfection by focus assay performed on 24-well plates in both mammal (Vero) and insect (*A. Albopictus*, C6/36) cells. Foci were visualized immuno-histochemically. Mutant viruses able to produce foci of infection were scored as positive (+) or negative (-) after 10 days of infection. Virus mutants giving foci in both hosts were amplified and titered to determine phenotype. AA; amino acid.

### Analysis of the DV2 deletion mutants

The transmembrane domains of membrane proteins are predicted to exist as α-helices within the membrane bilayer [22]. This structure is preferentially adopted due to the lack of water in the lipid bilayer resulting in greater stability of the TMD side chains in a helical structure [23]. This stability results from the ability of the carbonyl oxygen of each residue to form hydrogen bonds with the backbone HN of every fourth residue along the helical vertical axis [24]. While the exact geometry of the helix depends on the sequence there are basic physical properties of an ideal α-helix. There are 3.6 residues per helical turn which is a rotation of 100 degrees/residue, or one complete turn of 360 degrees. Thus, depending on the size of the TMD the relative position of the amino and carboxyl terminal ends will vary along the vertical helical axis. For the purposes of this discussion the angles which are referred to are the smallest angles either to the right or left direction with reference to the amino acid at the origin and the carboxyl terminus.

As a working model the sequence _452_SWTMKILIGVIITWIG_467 _(16 amino acids) was chosen as the E1 transmembrane region to be targeted for mutagenesis (Table 1). The sequence was determined based on previous studies, the present work and also on independent hydrophobicity plot analyses [3,18,21,25]. In the 34 mutants constructed certain trends were observed. Deletions were better tolerated when the viruses were grown in Vero cells. Because the size of the deletion was not directly related to the ability of the mutant to produce virus from these cells (Δ1-5 amino acids), membrane thickness did not appear to be a sole factor in production of most of the mutants. Certain mutants did not grow in either mammalian or insect cells (see table 1). One deletion of five residues which did not produce virus in either cell line was Δ_457_ILIGV_461. _Other five amino acid deletions were tolerated in Vero cells suggesting that five residues can be deleted; however, the region _457_ILIGV_461 _is critical for virus production from both Vero and C6/36 cell lines. Because LIG and GVII can be deleted and produce host-range mutants favouring growth in insect cells the region LIGVII may be important for interactions of the E TMD. A second mutant, ΔK_456_**,**was not tolerated in either cell line suggesting that this amino acid is important for virus production in both cell lines or that this position in the helix has specific structural requirements since other single amino acid deletions did produce virus from both hosts. The deletion of M_455 _also abrogated virus production from both host cells. This residue is adjacent to K_456 _which as a single deletion is also critical to expression in both host cells. When both residues are deleted (ΔMK_455-56_)virus production was restored only in Vero cells. Substitution of A for K_456 _restored virus production suggesting that structural requirements were re-established and that the amino acid K is not required at this position. A third mutant which did not produce virus in either cell line was ΔIIT_462_-_64_. This phenotype could be due to a critical distortion in the geometry of the ΔIIT_462_-_64 _TMD because ΔT and ΔIT are able to produce virus in Vero.

while Vero cells were generally more tolerant than the C6/36 cells to disruptions in the E1 TMD there were some differences seen in the C6/36 expressed TMD deletion mutants. I_463 _is a required residue for virus production in C6/36 cells. Since other single amino acid deletions were tolerated in these cells, again this result could be explained by the requirement for a specific amino acid at this position or disruption of the necessary helical geometry at this residue. Because a second I_462 _is available to replace the I_463 _deletion it is possible that the necessary geometry is affected in this mutant. It is of interest that the deletion of I_463 _is included in the ΔGVII which restores virus production in C6/36 cells. A deletion of four amino acids would approximate a full helical turn and restores the ability of ΔGVII to produce virus lost by the deletion of I_463_. As would be expected for a virus with such a wide host range, one mutant ΔG_460 _displayed a phenotype which was also a host-range mutant but was selective for Vero cells. Notably, no two or five amino acid deletion mutants produced virus in C6/36 cells. This was not the case for double deletion mutants grown in Vero cells possibly due to the greater flexibility of the mammalian membrane and the immediate lipid environment. For the mosquito cells a two amino acid deletion is the most disruptive deletion to the orientation of the NH terminus with respect to the COOH terminus placing the backbone of the termini 140 degrees from one another normal to the central helical axis. The predicted geometry for this wild type model sequence is used as an example and places the location of the NH and COOH termini within 60 degrees from the origin of the helix (the NH terminus located on the luminal side of the membrane). Thus, in E mutants' where the helical geometry contains a double deletion, a 140° angle of entry to exit from the membrane of these mutants did not produce virus in C6/36 cells. Also, there are no mutants that delete I_457_, or T_464 _capable of growth in insect cells making these essential residues or locations. It is of note that while W_465 _is completely conserved among DV2, its' deletion was tolerated in Vero cells. Motifs such as these may be host adaptive sites required for function in one specific host but not the other. Taken together these data identify the region or sequence _458_LIGVII_463 _as the region of the TMD which can be mutated to produce host-range mutants.

The collection of mutants made in the DV2 M protein is shown in Table 2. The TMDs of both glycoproteins were targeted for mutagenesis because one host-range mutant in Sindbis virus E1 was identified (Ribeiro, M *et.al. *in preparation). While the M and E domains cannot be directly compared because of the difference in size (15 amino acids compared to 16 in the E1 model) general observations can be made. With the exception of mutants 7 and 8 (Table 2), production of mutant virus was seen from both cell lines. However, no host-range mutations were identified in the T-1 region of M.

**Table 2:** Dengue virus deletion mutations produced in the first TMD of the M protein of DV2

SEQUENCE M-T1 DOMAIN			Foci		Critical AA Vero	Critical AA C6/36
					
			Vero	C6/36		
	**WT DV2**	**_245_PGFTMMAAILAYTIG_259_**	**++**	**++**		

1	ΔA_251_	**PGFTMM-AILAYTIG**	**+**	**+**		

2	ΔAA_251-52_	**PGFTMM--ILAYTIG**	**-**	**-**	AA_251-52_	AA_251-52_

3	ΔAAI_251-53_	**PGFTMM---LAYTIG**	**-**	**-**	AAI_251-53_	AAI_251-53_

4	ΔAAIL_251-54_	**PGFTMM----AYTIG**	**+**	**+**		

5	ΔAAILA_251-55_	**PGFTMM-----YTIG**	**-**	**-**	5aa	5aa

6	ΔM_250_	**PGFTM-AAILAYTIG**	**+**	**+**		

7	ΔMM_249-50_	**PGFT--AAILAYTIG**	+	-		M_249_

8	ΔTMM_248-50_	**PGF---AAILAYTIG**	+	-		M_249_

9	ΔFTMM_247-50_	**PG----AAILAYTIG**	**-**	**-**	5aa	5aa

The primary DV2 mutant focus assay screen determined which mutants were able to produce foci of infections in Vero and *A. Albopictus *C6/36 cells. (+) indicates the presence of foci and (-) indicates no foci were observed. AA; amino acid.

Thus, two host range deletion mutants predicted by the original hypothesis were identified in the E1 TMD and were selected for further analysis. While the initial hypothesis was that host-range phenotypes would be found among the larger mutants which could only properly assemble in the thinner insect membranes, it is clear from these results that the expression of a host-range phenotype is not only a function of the size of the deletion, but also of the helical geometry within the membrane, the membrane composition and by extension its effects outside the membrane. This methodology will be extended to look for equivalent host-range mutants within the corresponding regions of other serotypes of Dengue 1, 3 and 4 which may follow a similar pattern of expression. The importance of the E1 TMD deletion structures reported herein will be further discussed in terms of two desired phenotypes. The deletion mutants screened and selected for further study were determined by their ability to 1) produce host-range mutants with preferential growth to the insect host and 2) induce a neutralizing antibody immune response in a mouse model.

### DV2 mutants with deletions in the TMD of E or M proteins make normal viral protein and infectious virus

We have identified a region of the DV2 E-T1 domain which when removed yields a host-range phenotype in which virus production/infectivity is shifted to favor production in insect vs. mammalian cells. To date, 43 independent mutations have successfully been produced in the full-length DV2 clone (Tables 1 and 2). All DV2 E-T1 and M-T1 deletion mutant clones were confirmed by sequence analysis (Eurofins, MWG Operon).

A preliminary DV2 mutant screen was used to determine which DV2 mutant clones were able to produce infectious centers (foci). This screen is a direct plating of C6/36 and Vero cells transfected with transcripts of each mutant virus. These results did not reveal the host-range phenotype because titers were not calculated; instead the mutants were scored positive or negative depending on the presence or absence of foci. These results determined which mutants continued to the next screen (phenotypic analysis).

Analysis of the focus assay results confirmed the importance of the E-T1 and M-T1 domains in the assembly and expression of DV2. The focus assay enabled the visualization of infectious centers in the cells infected by the viral RNA. Based on number and size of foci, every mutant, even the single amino acid deletions, had reduced expression in both Vero and C6/36 cells. Twelve DV2 deletion mutants [9 in E-T1: DV2ΔG_460_, ΔV_461_, ΔW_453_, ΔT_454_, ΔLIG, ΔGVII, Δ ILIG, ΔILI and ΔIGV (Table 1) and 3 in M-T1: DV2ΔA, ΔAAIL, and ΔM_250 _(Table 2)] capable of producing foci of infection in both C6/36 and Vero cells were identified. Five mutants expressing the best titers (E-T1 mutants DV2ΔG, DV2ΔLIG, ΔIGV, and ΔGVII; M-T1 mutant DV2ΔAAIL) were selected to proceed to the secondary screen.

### Deletion mutants constructed around a specific TMD position/region exhibit host-range phenotype

Five mutant viruses from the primary screen underwent a second round of screening to ascertain phenotype. The mutants were transcribed into RNA and transfected into the preferred host, C6/36 cells. The mutant viruses were then grown in both Vero and C6/36 (at the same starting MOI of ~0.03 ffu/cell) to determine growth characteristics in mammalian vs. insect cells. Virus was harvested on day 7 and titered on Vero cells. The foci formed by these mutants were very small and were visualized using a more sensitive fluorescent focus assay (described in Methods). Mutant viruses with preferential growth in the C6/36 mosquito cell line and attenuated growth in Vero cells as defined by at least 2 orders of magnitude less virus production in Vero were considered to express the host-range phenotype.

**Figure 2:**
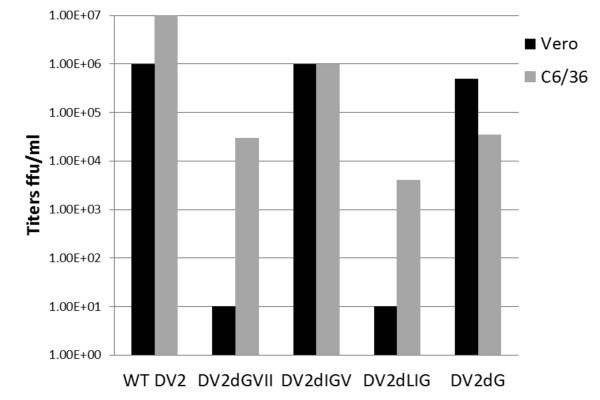
**Host-range phenotype of DV2 mutants. Titers for the WT DV2 and mutant viruses are shown**. All virus strains were grown in Vero (mammal; black bars) and the C6/36 (insect; gray bars) cells to measure host-range phenotype. The titers of the mutant viruses were determined by a fluorescent focus assay. Titers of WT virus were measured by a colorimetric focus assay. DV2ΔIGV did not display the host range phenotype and DV2ΔG was a host range mutant which grew better in the Vero cells. Only DVΔGVII and DVΔLIG were chosen for further analysis.

Out of 5 mutants that produced foci in both C6/36 and Vero cells and were chosen to proceed to the secondary screen, only 2, DV2ΔLIG and DV2ΔGVII, showed the host-range phenotype restricted to preferential growth in the insect cells (Figure 2). DV2ΔIGV grew equally well in both cell lines (Figure 2). The opposite host-range phenotype, favoring growth in mammalian cells was observed for the DV2ΔG mutant (Figure 2). Upon further passage, the M-T1 domain mutant DV2ΔAAIL did not yield measurable titers in either Vero or C6/36 cells (data not shown).

WT DV2 routinely generates titers of 10^6 ^ffu/ml in Vero cells and 10^7 ^ffu/ml in C6/36 cells. It was observed that most mutants gave lower titers as compared to WT DV2, except DV2ΔIGV, which was quantitatively identical to WT in Vero cells, but was qualitatively (i.e. foci were much smaller) reduced as compared to WT, as was observed for DV2ΔLIG and DV2ΔGVII as well. Titers were in the range of 10^3^- 10^4 ^ffu/ml for both DV2ΔGVII and DV2ΔLIG mutants grown in C6/36 cells (Figure 2).

### Protein expression and processing of DV2 host-range mutants

**Figure 3:**
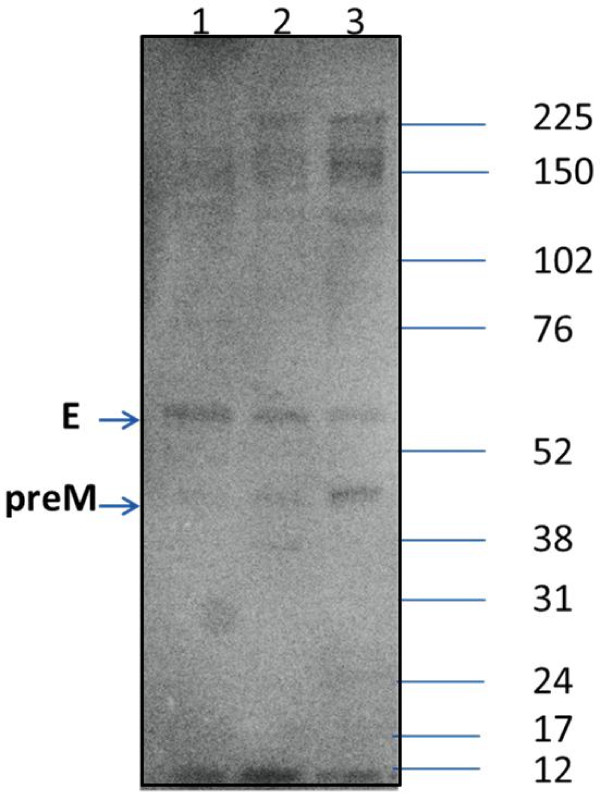
**Western blot analysis of DV2 host-range mutants**. A 4-12% bis-tris gradient gel was loaded with 5 μl of iodixanol-gradient purified WT or 25 μl of mutant DV2 virus purified from the supernatant of infected mosquito C7-10 cells. Proteins were stained using a polyclonal serum raised against whole DV2. The size of the molecular weight markers is indicated on the right in kDa. Lane 1, WT DV2; Lane 2, DV2ΔGVII; Lane 3, DV2ΔLIG. E; E protein; preM, preM protein.

The two DV2 host-range mutants DV2ΔGVII and DV2ΔLIG were found to produce infectious virus, as observed in the focus assay (Table 1 and Figure 2). In order to determine if all viral proteins were indeed produced and processed as in WT DV2, a western blot analysis was performed (Figure 3). Virus grown in mosquito cells was harvested from the cell supernatant at day 7 post-infection and examined by SDS-PAGE. Equal protein amounts from each virus were added to the gel. To confirm the presence of specific DV proteins, the gel was transferred to substrate and blotted with an anti-DV whole virus antibody. Visualization of the protein bands revealed a similar banding pattern to that of WT DV2 (Figure 3) showing preM and E, verifying the correct production and processing of virus proteins by the mutants.

### Electron micrographs of the mutants (DV2ΔGVII and DV2ΔLIG) suggest impaired assembly

**Figure 4:**
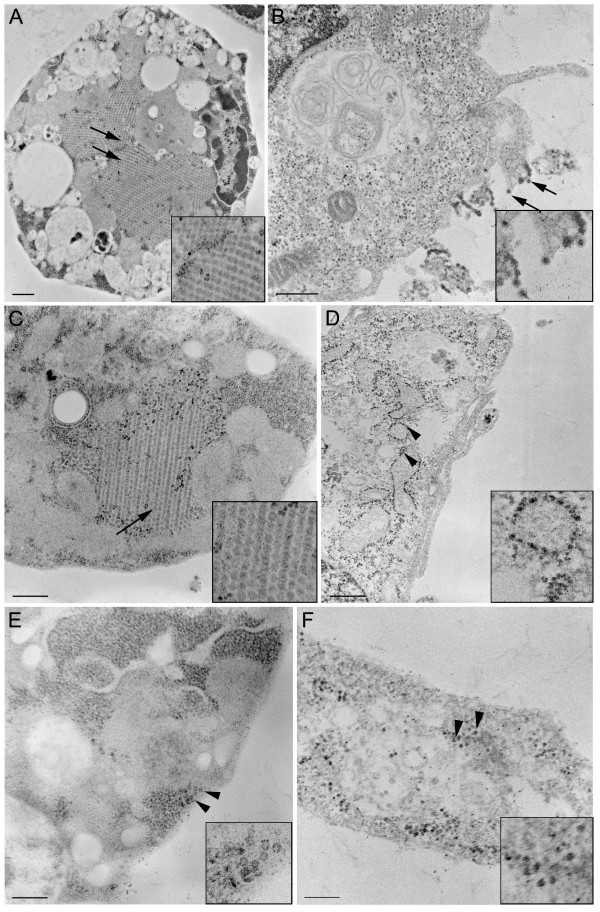
**Electron micrograph thin sections of WT DV2, DV2ΔGVII, DV2ΔLIG viruses in the C6/36 line of mosquito cells (column 1; frames A, C, & E) and the mammalian Vero cell line (column 2; frames B, D, & F). WT DV2 virus particles are seen in large paracrystalline structures in infected mosquito cells (Figure 4A-arrows and inset)**. In infected mammalian cells, WT virus particles are associated with the plasma cell membrane (Figure 4B-arrows, inset). Mosquito C6/36 cells infected with the DV2ΔGVII mutant display similar amounts of virus particles (Figure 4C-arrows, inset) as compared to that observed in the WT virus infected mosquito cells. However, in mammalian cells infected with DV2ΔGVII (Figure 4D) only the presence of nucleocapsids in the cytoplasm and associated with internal membranes were detected in the thin sections (arrowheads and magnified in the inset). Similarly, DV2ΔLIG virus particles were observed in the cytoplasm of infected C6/36 mosquito cells although fewer particles were observed (Figure 4E-arrowheads) while only nucleocapsids were detected in the cytoplasm of the infected mammalian cells (Figure 4F-arrowheads, inset). Bars are 500 mM and the insets are 2X magnified from the original.

In order to analyse the differences in virus ultrastructure between mammal and mosquito cells, thin sections of cells infected with WT and mutant viruses were prepared and evaluated. C6/36 (Figure 4A) and Vero (Figure 4B) cells were infected with the WT DV2. Virus particles were observed in large paracrystalline structures within the mosquito cell (4A-arrows) and associated with the mammalian plasma cell membrane (4B-arrows). In Figure 4C, mosquito cells infected with the DV2ΔGVII mutant exhibited similar amounts of virus production as compared to WT (arrows). However, in mammalian cells infected with DV2ΔGVII (Figure 4D) only the presence of nucleocapsids in the cytoplasm could be detected in the thin sections (arrowheads) suggesting a defect in assembly, budding and release of virus into the supernatant. Cells infected with the mutant DV2ΔLIG displayed a similar phenotype. The presence of virus particles was observed in the cytoplasm of mosquito cells (Figure 4E-arrowheads) while only nucleocapsids were detected in the cytoplasm of mammalian cells (Figure 4F-arrowheads) [26]. These phenotypes of WT, ΔGVII and ΔLIG were detected in 90%, 70% and 25% of observed cells respectively. These observations clearly demonstrate differences in virus production between cell types as expected for host-range mutants. The specific defect in the assembly pathway of these mutants in Vero cells is not known. It is hypothesized that one aspect of the disrupted assembly would be the inability of the E-T1 domain to integrate properly into the membrane of the thicker mammalian ER producing a host-range mutant as was seen in the distantly related Alphavirus Sindbis [11]. While much progress has been made in solving the molecular mechanism of flavivirus assembly, it has yet to be established how the mature particles are formed [27,28]. However these results clearly demonstrate differences in virus assembly between the cell types as expected for host-range mutants.

### Mutants with host-range phenotypes generate neutralizing antibody response in BALB/cJ mice

**Figure 5:**
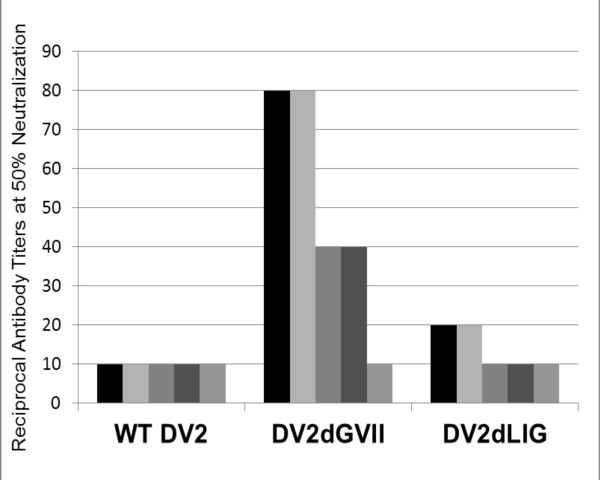
**Murine neutralizing antibody titers were measured by the focus reduction neutralization assay (FRNT) for mice injected with WT DV2, the mutants DV2ΔLIG and DV2ΔGVII, and iodixanol in PBS-D (mock)**. All assays were performed on Vero cells. Individual mouse serum from each of the five mice in each experimental group was analysed and the titers shown as the reciprocal neutralization titer at 50% neutralization. Each bar represents one mouse. Mice were injected with 29 μg of each virus, equivalent to 2 × 10^2 ^ffu WT DV2, 4 × 10^2 ^ffu DV2ΔGVII, or 2.4 × 10^3 ^ffu DV2ΔLIG. Mice injected with the mock inoculum produced no measurable neutralizing antibody.

The goal of the subsequent mouse trial with the DV2 host-range mutants was to determine the *in vivo *immunogenicity of each mutant. Previous research has concluded that the murine model (BALB/cJ) is not amenable for DV2 pathogenesis [29-32] but is useful for the determination of antibody responses to dengue vaccine candidates [3,33-36]. The rationale for this study was to determine if any antibody response could be detected in an animal model other than non-human primates. Mutants possessing an insect cell preferential host-range phenotype were analyzed for the ability to generate neutralizing antibodies in BALB/cJ mice. The subcutaneous route of infection was employed in order to approximate a physiologically relevant DV transmission by mosquitoes. Gradient purified host-range mutant virus was used as an inoculum. 29 μg of protein in 100 μl of iodixanol in PBS-D, equivalent to ~10^2 ^to 10^3 ^total ffu was injected for each vaccine candidate and WT DV2 control. As a negative control, iodixanol buffer alone was injected into one mouse group. Each inoculum was quantified using the EZQ^® ^Protein Quantitation Kit. The amount (29 μg) was chosen based on the yield of the lowest titered virus (DV2ΔLIG) to ensure all mice received equal doses. For wild-type virus this was accomplished by using a low titer DV2 stock. Both mutants were able to generate reciprocal antibody titers at 50% virus neutralization (ND_50_) in the range of 10-80 ND_50_. Mice inoculated with DV2ΔGVII produced a higher neutralizing antibody response (80 and 40 ND_50_) as compared to those who received DV2ΔLIG which gave a response of 20 and 10 ND_50 _or WT DV2 at 10 ND_50_. Four out of 5 mice in the DV2ΔGVII group gave an ND_50 _between the range of 20-80 while only 2 mice were responders in the DV2ΔLIG group, with an ND_50 _of ~20 (Figure 5). Mice injected with buffer alone exhibited no neutralizing antibody response. RT-PCR analysis of amplified murine serum samples taken at the conclusion of this study yielded no detectable virus indicating that the virus inoculum and any subsequent replicating virus was cleared. Further studies on the immunogenicity of these mutants and their utility as vaccine candidates will be conducted in non-human primates.

### Mutant viruses grown in C6/36 cells are stable and do not revert to wild type virus

The host-range mutant viruses identified in this study propagate two orders of magnitude less virus than the WT virus in insect cells. These host-range mutants, however, still produce two to three orders of magnitude less infectious virus from Vero cells as compared to C6/36. As a result, there may be some selective pressure on the mutant viruses to revert to the WT virus in the insect system. To confirm whether or not this was the case, reversion of the mutant viruses to WT in the C6/36 cells was evaluated over five serial passages. The cell culture supernatant was collected from the infection after each round of infection and the sequence of viral RNA was analysed by RT-PCR. The mutant viruses retained their deletions after 5 sequential passages in C6/36 cells and no reversion to the WT DV2 sequence was found. The same results were obtained when the experiment was performed in mammalian Vero cells. After 4 serial passages in the Vero cells, no WT virus or reversions were recovered from the mutant virus infected cells.

## Discussion

In nature, arboviruses cycle between vertebrate and invertebrate hosts in a complex life cycle. Vertebrate and invertebrate membranes express divergent biochemical and biophysical membranes of distinct physiology. This difference has imposed on the arbovirus genome the necessity to adapt to a vast divergence during its life cycle. Taxonomically the Class *Insecta *is defined as a group of cholesterol auxotrophs [37]. This characteristic is an important distinction because cholesterol alters the properties of membranes making them thicker, more viscose and ion impermeable. A hypothesis was previously put forth which predicted that alphavirus host-range mutants with preferential growth in the invertebrate host could be constructed by producing deletions in the TMD of the virus glycoprotein(s). This theory was first tested and supported in the alphavirus Sindbis (SV) [11]. It was further postulated that this property of differential glycoprotein requirements for TMDs anchored in the thinner and biochemically distinct insect membranes would apply to all arboviruses. While two host-range mutants of DV2 were characterized in this study, analysis of a series of deletions in the E-T1 and M-T1 domains of DV2 revealed a more complex relationship between the transmembrane domain and lipid bilayer. Other factors such as helical conformation and interactions of specific amino acid residues play a critical role in TMD function in each host.

Two significant contributions of this study to the field of arbovirology are reported. First, data are presented which identifies a region in the T1 domain of DV2 E, in the sequence aa _458_LIGVII_463 _which is essential for virus assembly and infectivity in mammalian cells, but is not required in insect cells, as predicted from the SV model system. The membrane of the host cell is one critical structural common denominator which the alpha and flaviviruses have in these studies. It is of interest that only the E protein produced host-range mutants while M did not. It may be concluded from these observations that M does not contain a TMD motif which can define host-range but that it is of major importance in the formation of infectious virions. While the E protein did display a region which could be deleted to produce host-range mutants restricted to growth in the insect hosts, this observation suggests that M and E have unique roles in virus assembly.

Second, both DV host-range mutations identified produced significant numbers of non-infectious virions in the mosquito system. This was determined by measuring the particle to pfu ratios of all these strains. WT DV2 has a particle to pfu ratio of 10^3 ^particle/pfu while ΔGVII and ΔLIG have particle to pfu ratios in the 10^6-7 ^particles/pfu range. This feature was a phenotype also associated with the SV deletion mutants. The presence of non-infectious particles in the vaccine strains could present different epitopes or act as adjuvant. A significant difference in the assembly of alpha and flaviviruses is the association of the glycoprotein-modified viral membrane with the nucleocapsid. Alphaviruses are characterized by the strong association of the E2 tail with the nucleocapsid which is required for assembly and infectivity [38]. The flaviviruses do not directly interact with the nucleocapsid and the mechanism by which virus budding occurs in association with the core is not well understood [39,40]. Additionally, flaviviruses produce empty particles [40-42] which increase toward late stages of infection (M. Ribiero, R. Hernandez personal observation) suggesting that some component (viral or host) is depleted as the infection progresses. These specific differences in the details of virus assembly in the alpha and flavivirus systems underscore the importance of the membrane in the host-range phenotype. It is for this reason that it is expected that this technology for the development of arbovirus vaccines can be applied to other flaviviruses and alphaviruses.

The ability of DV to assemble host-range mutants in insect cells was employed to test for DV vaccine strains in mice. Although mice are not a model host for Dengue virus, this experiment was performed as a test of the immunogenicity of selected host-range mutants prior to study in a non-human primate model. Indeed, the host-range mutants [43,44] were found to illicit high neutralizing Ab compared to the wild-type control when tested in BALB/cJ mice. DV2ΔGVII was found to elicit a stronger neutralizing response in 4 out of 5 mice tested while the DV2ΔLIG mutant showed a response in 2 of 5 mice injected. This differential response between the mutants and WT, as well as between the 2 mutants themselves, was not an unexpected result; this phenomenon was also observed in the SV system (5). These results indicate that although E is truncated in the TM domain, the E antigen is still presented to the host immune system in an immunogenically competent conformation exceeding that seen in the wild-type infection. Altering the length and composition of the E-T1 α-helix may lead to changes in the conformation of that helix that are transduced to the extracellular domain of the E protein. Therefore, while all extracellular epitopes of the host-range mutants are WT in sequence, they may have an altered conformation that allows for better access to existing or hidden neutralizing antibody epitopes, leading to a more robust and varied NAb response as compared to WT. It will be of interest to determine if this enhanced neutralizing effect is the result of a combination of altered immunogenic factors presented by these mutants which engages the immune system in an alternate pathway than other previously tested live attenuated vaccine strains. More detailed studies of the immunogenicity of these mutants are on-going. Of note is that DV2ΔGVII, which is the larger of the two deletion mutants (i.e. the E-T1 domain has fewer amino acids) elicited six fold more neutralizing activity than the smaller DV2ΔLIG deletion mutant. Although mice are not normal hosts for DV, this study was done solely to test the ability of these mutants to induce an immune response in mice prior to further study. Other facets of the immune response to these mutants will be evaluated in a more suitable primate model. Although non-human primates are also insufficient to predict vaccine safety, immunogenicity and efficacy [43,45,46] monkey studies will determine if these mutants meet the criterion to be tested in humans [47,48].

It is expected that these types of mutations can be constructed for DV1-4 and used to formulate a tetravalent vaccine. This study describes a novel approach to DV vaccine development in which only molecular biology methods are required for production of a vaccine strain. This combination of host-range mutants for DV1-4 would constitute a live virus vaccine grown in insect cells. The DV host-range mutants were shown not to revert to WT after multiple passages, displaying genetic stability in the host used for production. The mechanism of genetic stability of these host-range mutants in insect cells is not known, but alphavirus complementation does not occur in insect cells implying component sequestration [5,49]. Further, although these viruses exist as quasi-species due to the high error rate of RNA polymerase; this did not affect the stability of the deletion over five passages *in vitro*. These data provide further evidence that sequence elements which define host-range are expressed in the arbovirus glycoprotein TMDs and probably throughout the arbovirus genome. This methodology will continue to be applied to other pathogenic flavi and alphaviruses to produce vaccine strains.

## Conclusions

Deletions in the E-T1 TMD of DV2 were created that produced an altered host-range phenotype favoring growth in insect hosts. Factors other than TMD length alone were also found to influence the phenotype of DV2 mutants. The E domain sequence _458_LIGVII_463 _contains motifs responsible for host specificity. Targeting arboviral TMDs to create host-range mutants is a novel method of producing potential live vaccine candidates and may be applied to many insect-transmitted viruses.

## Abbreviations

C: Capsid; DV2: Dengue Virus serotype 2; DV: Dengue Virus; E: Envelope, EM: Electron Microscopy; ffu: Focus Forming Unit; TMD: Transmembrane Domain; MOI: Multiplicity of Infection; NAb: Neutralizing Antibody; PCR: Polymerase Chain Reaction; FRNT: Focus Reduction Neutralization Test; SC: Subcutaneous; SV: Sindbis Virus; WT: Wild-Type.

## Competing interests

The authors declare that they have no competing interests.

## Authors' contributions

RH, DTB, KN, and KMS conceived and designed the experiments. KN, KMS, CJS, MR, AP and GST performed the experiments. RH, DTB, KMS, KN, AP and MR analyzed the data. RV performed all electron microscopy studies. RH, KMS and KN wrote the paper. MET provided experimental equipment/supplies and helped coordinate and design the studies. All authors read and approved the final manuscript.
